# *MapsTorch*: automatic differentiation for X-ray fluorescence data analysis

**DOI:** 10.1107/S160057752501032X

**Published:** 2026-01-01

**Authors:** Xiangyu Yin, Zichao Di, Olga Antipova, Si Chen, Yi Jiang, Arthur Glowacki

**Affiliations:** a Advanced Photon Source Argonne National Laboratory Argonne IL60439 USA; Paul Scherrer Institut, Switzerland

**Keywords:** X-ray fluorescence, automatic differentiation, machine learning, optimization

## Abstract

X-ray fluorescence (XRF) is a popular spectroscopy technique for elemental analysis. Spectrum fitting and parameter tuning are at the core of XRF analysis and are conventionally manually intensive, especially for synchrotron experiments involving large amounts of diverse samples. This work introduces the automatic differentiation (AD) technique to XRF and an open-source package called *MapsTorch*. By transforming an analytical model of the XRF spectrum into a differentiable computation graph with AD, *MapsTorch* enables robust optimization of parameters and elemental intensities. We evaluate *MapsTorch* by conducting computational experiments on a large number of historical synchrotron XRF datasets and compare its performance with the currently practiced fitting tool *NLopt*. The results show that *MapsTorch* consistently achieves high-quality fits and often leads to better fitting quality than *NLopt*, particularly in tasks such as initial spectrum fitting and elemental intensity refinement. The robust performance of *MapsTorch* paves the way for developing automated and high-throughput XRF data analysis workflows to handle the increasing data volumes expected from next-generation synchrotron facilities.

## Abstract 2

*MapsTorch* is a new open-source package that enables automatic differentiation for X-ray fluorescence (XRF). The robust performance of *MapsTorch* makes it possible to automate steps that are usually manual in XRF analysis, including initial spectrum fitting and elemental intensity refinement, and therefore can support automated, high-throughput workflows for synchrotron facilities.

## 1. Introduction

X-ray fluorescence (XRF) is an analytical technique used to determine the elemental composition of materials. When a sample is irradiated with high-energy X-ray beams, electrons in the inner shells of atoms are excited and ejected. As electrons from higher energy levels fill these vacancies, fluorescent X-rays with characteristic energies are emitted. By measuring and analyzing the energies and intensities of these fluorescent X-ray photons, researchers can identify and quantify the elements present in a sample. XRF spectroscopy offers many advantages, including non-destructive analysis, minimal sample preparation, and the ability to detect a wide range of elements simultaneously (Margui & Van Grieken, 2013 ▸). The technique is applicable to solid, liquid, and powdered samples, making it widely adopted across a range of applications (Melquiades & Appoloni, 2004 ▸; Liritzis & Zacharias, 2011 ▸; Uo *et al.*, 2015 ▸; Oyedotun, 2018 ▸; Feng *et al.*, 2021 ▸).

XRF has become widely used in synchrotron facilities. The high brilliance and tunable energy of synchrotron radiation further enhance the capabilities of XRF spectroscopy, enabling trace element analysis and chemical speciation (Bertsch & Hunter, 2001 ▸; Mino *et al.*, 2018 ▸). Furthermore, by raster scanning a focused X-ray beam across a sample and collecting XRF spectra at each point, users can generate two-dimensional or even three-dimensional maps of elemental concentrations (Chen *et al.*, 2014 ▸; Chen *et al.*, 2015 ▸). This capability enables visualization and quantification of elemental gradients, identification of localized features, and correlation of elemental distributions with other sample properties. For example, in materials science, XRF mapping has been used to study the distribution of dopants in semiconductors (Troian *et al.*, 2018 ▸), analyze the composition of multilayer thin films (West *et al.*, 2017 ▸), and investigate elemental segregation in alloys (Feng *et al.*, 2020 ▸). In environmental science it has been applied to track pollutants in soil and sediment samples (Manceau *et al.*, 2002 ▸), while in biology it has enabled the visualization of metal accumulation in tissues and cellular structures (Munro *et al.*, 2008 ▸). Moreover, the non-destructive nature of XRF mapping is particularly valuable for precious or unique samples, such as cultural heritage artifacts or rare geological specimens (Cotte *et al.*, 2018 ▸).

With the increasing volume and diversity of XRF samples in synchrotron facilities, automating XRF data analysis has become a goal. For example, a typical XRF mapping experiment at a synchrotron facility can generate thousands of spectra in a matter of hours, making manual intervention impractical and thus calls for automated XRF data analysis workflows. Traditional XRF data analysis depends on manual parameter tuning via iterative spectrum fitting to obtain an instrument and dataset specific parameter configuration. The tunable parameters in XRF fitting include energy calibration coefficients, detector response parameters, peak-shape function coefficients, *etc*. This manual tuning process is time consuming, can introduce user to user variability, and cannot be automated and scaled to high throughput experiments where large amounts of data are collected.

**Figure 1:**
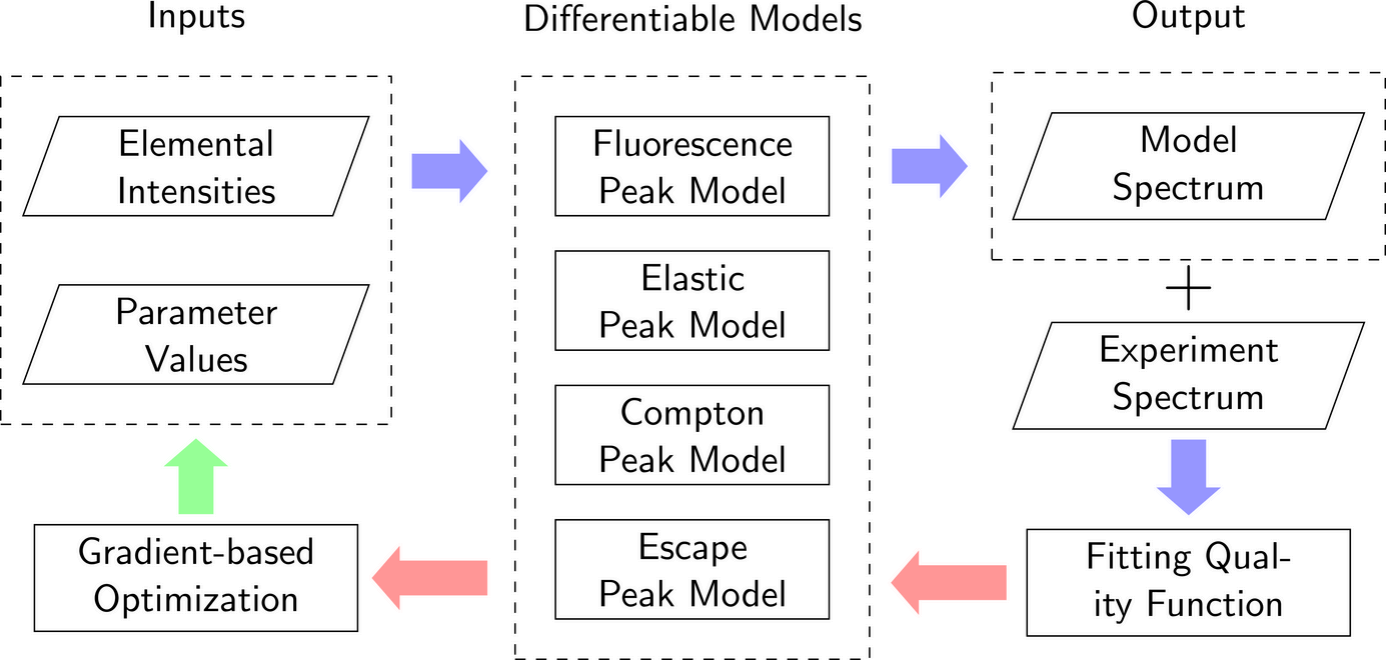
Schematic of the differentiable computational graph in *MapsTorch*. Inputs include elemental amplitudes and fitting parameters. The differentiable models include fluorescence peak lines, elastic and Compton scattering, and escape peaks. The sum (along with background) forms the model spectrum (Output), which is compared with the experimental spectrum via a loss/fitting quality function. AD supplies gradients to an optimizer such as Adam, closing the optimization loop. Blue arrows mean the forward process, red arrows mean the AD gradient calculation process, and the green arrow means the optimization process.

In this study we apply automatic differentiation (AD) to tackle this challenge. AD is a modern computational technique that has been widely adopted in the machine learning and optimization communities in the last decade (Baydin *et al.*, 2018 ▸). AD provides a systematic way to compute gradients of complex functions more efficiently and with higher accuracy, compared with traditional numerical methods. Our open source package, *MapsTorch*, implements the classic *MAPS* analytical models (Nietzold *et al.*, 2018 ▸; Vogt *et al.*, 2003 ▸) using the *PyTorch* framework (Paszke *et al.*, 2019 ▸) to enable AD, as illustrated in Fig. 1 ▸. This differentiable implementation unlocks advanced AD-based optimizers which show superior performance in our computational experiments for XRF spectrum fitting tasks that are conventionally manually intensive.

To summarize our contributions, we recasted the classic *MAPS* analytical models as a fully differentiable computation graph using *PyTorch* and introduced an open source tool called *MapsTorch*. This new implementation enables joint optimization of fitting parameters and elemental amplitudes with advanced AD-based solvers. We carried out computational experiments on approximately 1200 historical datasets and demonstrated robust performance of *MapsTorch* for (1) first-pass fits without good parameter initialization and (2) elemental intensity refinement under tuned parameters. These results indicate that *MapsTorch* can effectively minimize human effort and handle the scale and variability in future automated synchrotron XRF workflows.

## 2. Methods

### 2.1. XRF analytical model

XRF data analysis is based on an analytical model of the XRF spectrum. The typical analytical XRF spectrum model consists of contributions from various components (Van Grieken & Markowicz, 2001 ▸). The main contributing components are the characteristic fluorescence emission line peaks. These peaks are commonly modeled using Gaussian profiles,
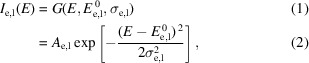
where *I*_e,l_(*E*) represents the intensity at energy *E* for a specific characteristic line l (*e.g.K*_α_, *K*_β_) of a specific element e. *A*_e,l_ is the peak amplitude, *G* is the Gaussian function, 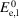 is the peak center (*i.e.* characteristic emission line energy), and σ_e,l_ determines the peak width.

In addition to the fluorescence line peaks, other contributing components include the elastic (Rayleigh) scattering peak *I*^R^(*E*), inelastic (Compton) scattering peak *I*^C^(*E*), escape peaks 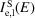, *etc*. The complete model spectrum is the sum of all these components,
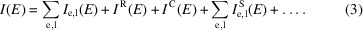
In practical XRF data analysis, the analytical model is further adapted. For example, the Gaussian peak shape is extended with step and tail functions to reflect detector behavior. A background spectrum is estimated from the experimental data and is added to the model to account for baseline and noise contributions. For example, the classic Statistics-sensitive Nonlinear Iterative Peak-clipping (SNIP) algorithm (Ryan *et al.*, 1988 ▸) iteratively replaces each channel by the minimum of its current value and the average of symmetrically offset neighbors over increasing window sizes until the continuum is obtained. Besides, predefined branching ratios are often used to regulate intensities of different lines for the same element. With the modified model spectrum *I*^r^, we can then fit an experimental spectrum *I*^expr^, essentially solving an optimization problem, 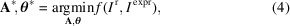
where **A** represents all peak amplitudes (*i.e.* intensities) and **θ** denotes all parameters in the spectrum models. *f* is an objective function that describes the fitting quality. For example, in the classic *MAPS* model, **θ** includes the energy offset and coefficients, coefficients of the peak shape model, step and tail parameters, Rayleigh and Compton scale factors, *etc*. Those parameters require adjustments for specific experiment setup and sample, which is time consuming and prone to inconsistency, especially under user facility settings.

### 2.2. AD and *PyTorch*

AD is a modern computational technique for numerically calculating gradients of complex functions. Unlike traditional methods, such as finite difference, that treats the function as a black box, AD leverages the function’s mathematical structure to calculate gradients. The key idea is to decompose the complex function into a computational graph of elementary operations (*e.g.* addition, multiplication, exponentiation) and basic functions (*e.g.*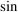, 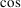, 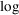), each of which has a known partial derivative. After constructing the computational graph, AD automatically applies the chain rule systematically across the graph to accumulate these partial derivatives.

In AD, gradients of a scalar loss (*e.g.* fitting quality metric) with respect to all parameters in the model can be calculated efficiently as multiples of single forward evaluations, whereas finite difference requires a number of forward evaluations proportional to the number of parameters and are sensitive to step sizes and noise. For the XRF spectrum analytical model that has many elemental amplitudes and fitting parameters inputs, AD can reduce gradient calculation cost, improve numerical stability, and remove the need to bookkeep derivative values. In practice, these features can further enable advanced AD-based optimizers to make coordinated updates that efficiently traverse the loss landscape, resulting in better optimization results.

In this work we enable AD with the *PyTorch* framework. The application of *PyTorch* in XRF immediately makes available advanced modern optimization algorithms. This is a practical advantage because these algorithms are designed to handle complex high dimensional problems efficiently, including those arising in large scale neural network training. For instance, the Adaptive Moment Estimation (Adam) optimizer (Kingma & Ba, 2014 ▸) that combines the advantages of Momentum and RMSprop techniques to adjust gradient updating rates works well with large and complex models and can often speed convergence and reduce sensitivity to step sizes. Here, ‘Momentum’ refers to keeping an exponentially decayed running average of past gradients to smooth updates and accelerate progress along consistent directions, while ‘RMSprop’ rescales each parameter’s step size using a running average of squared gradients to handle ill-conditioned problems. We refer readers interested in the optimization algorithm details to Tieleman (2012 ▸) and Sutskever *et al.* (2013 ▸). As the result, Adam often achieves strong results with minimal tuning and is a common default choice in *PyTorch*-based workflows. These advanced AD-based optimizers can overcome limitations of classical optimization approaches used in XRF analysis such as sensitivity to initial solutions and getting stuck in local minima.

AD and *PyTorch* also open up new possibilities for XRF data analysis. Because of the differentiability, one can customize the underlying analytical components easily without deriving gradients analytically or modifying the optimization loop. It is also possible to integrate the differentiable XRF spectrum model with broader data-driven models (*e.g.* deep neural networks predicting sample properties).

### 2.3. Objective and performance metric

As discussed in Section 2.1, a fitting quality objective *f* is needed. In this study, we use the mean squared error (MSE), which is the same objective used in the currently used fitting method *NLopt* (Johnson, 2007 ▸) for direct comparison. In synchrotron-based XRF measurements, total photon counts can vary dramatically; the resulting MSE values can also vary significantly. To compare fitting performance across datasets with vast different total photon counts, we use the coefficient of determination, *R*^2^, as our primary metric, which can be seen as a normalization of MSE. By definition, 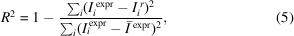
where 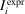 is the experimental spectrum intensity at energy channel *i*, 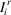 is the fitted intensity and 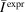 is the mean of the observed intensities. In the definition of *R*^2^, the numerator is effectively the MSE multiplied by the number of energy channels. By dividing by the total variance, *R*^2^ essentially normalizes the MSE on a per-spectrum basis, making it comparable across datasets with vastly different intensity scales rather than favoring high-count spectra. *R*^2^ is bounded by 1, which provides a straightforward and intuitive interpretation of the fitting quality and make it easy to compare. An *R*^2^ value of 1 indicates a perfect match between the fitted and observed spectra, while 0 or even negative values indicates that the model spectrum does not agree with the experimental spectrum at all.

We note that the Poisson-weighted statistic (*e.g.* χ^2^ variants or deviance) is another category of commonly used spectrum fitting objectives. The choice of using MSE instead of Poisson-weighted statistic at the Advanced Photon Source (APS) could be attributed to common preprocessing steps (dead-time corrections, background subtraction, and possible rebinning across instruments) that weaken the independent-Poisson assumption, the over-dispersion and correlations observed in real detector signals, the fact that MSE is computationally simple and robust across instruments, or simply historical practice persistence. To provide a like-for-like comparison with current practice, *MapsTorch* also optimizes MSE in this study. We acknowledge that Poisson-appropriate objectives are widely used in the community and can increase sensitivity to small peaks; evaluating, benchmarking and incorporating them within *MapsTorch* will be our next step.

### 2.4. *MapsTorch* implementation

The core logics of *MapsTorch* are implemented in the 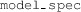 function. As illustrated in Table 1 ▸, the 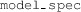 function calculates a model spectrum from input fitting parameters and elements, along with some other fitting configurations. Within the function, the 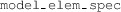 subroutine (Table 2 ▸) calculates an element’s spectrum, which consists of contributions from *K*, *L* and *M* lines, their relative intensities, and possible pileups. The peak shapes are modeled using a modified Gaussian function that includes tail and step features to account for detector effects. The 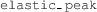 and 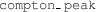 subroutines calculate the Rayleigh and Compton scattering peaks, respectively. The 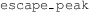 subroutine accounts for effects where characteristic X-rays from the detector material (*e.g.* Si) escape the detector, resulting in additional peaks at lower energies.

The use of *PyTorch* tensors and operations throughout the implementation ensures that all computations are differentiable and allows for efficient computation of gradients with AD. We then define a loss function (*i.e.* MSE) that quantifies the discrepancy between the model spectrum and the experimental data. After we compute the gradients of this loss function with respect to model inputs (*i.e.* intensities, parameters), we employ an advanced AD-based optimizer, such as Adam, to iteratively minimize the loss function and improve the fit between the model and the experimental spectrum.

The 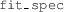 function (Table 3 ▸) implements an AD-based optimization loop for fitting parameters and elemental intensities simultaneously. Within the function, the 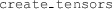 subroutine creates optimizable tensors for fitting parameters and peak amplitudes (*i.e.* elemental intensities), and initializes them using heuristics. The 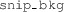 subroutine (Table 4 ▸) implements a differentiable SNIP background estimation algorithm. We note that the SNIP background estimate contributes to the loss and is part of the computational graph during back propagation.

### 2.5. User interface

*MapsTorch* gives users full control over the fitting process via Python API. For example, users can custom fitting settings by changing input arguments to 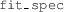. The 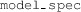 function along with other functions and objects can be used as standalone tools, such that it is possible to embed those functions and objects within other Python routines for higher level tasks like element selection or experiment control, which are beyond the scope of this work.

To facilitate learning of *MapsTorch* and streamline simple tasks via *MapsTorch*, we also implemented a lightweight graphical user interface via the *marimo* reactive notebook framework. For example, in the parameter optimization notebook, users can launch a *MapsTorch* fitting run that jointly optimizes fitting parameters together with elemental amplitudes. The reactive interface reports the live fit in linear and logarithmic scales and logs a history of candidate parameter configurations for comparison and rollback. Interested readers can try the interface with a demo dataset at https://huggingface.co/spaces/shawnyin/MapsTorch.

### 2.6. Related tools

We note that there are existing XRF data analysis tools such as *PyMCA* (Solé *et al.*, 2007 ▸), *PyXRF* (Li *et al.*, 2017 ▸) and *GeoPIXE*. According to their documentations or tutorials^1^ these tools rely on parameter file input, or interactive GUIs in which users can specify parameters in terms of energy calibration, peak shape, detector response *etc*. This design is well suited for experts to carry out exploratory workflows but is less compatible with automated processing workflows needed by synchrotron facilities to handle large and diverse user facility samples at scale. In contrast, *MapsTorch* expresses the spectrum model as a differentiable computation graph and jointly optimizes fitting parameters and amplitudes, which means that parameter inputs/files are optional. Thus *MapsTorch* has great potential in reducing human efforts and developing fully automated XRF data analysis workflows. We note that we do not intend to replace expert-driven analysis with other tools; instead we aim to reduce manual effort and enable scalable, consistent processing in automated processing settings. Besides, as mentioned in Section 2.5, *MapsTorch* provides fully customizable Python API, functions and objects, which can be integrated with other Python routines and serve as a building block embedded within other higher level automated workflows. Our computational experiments in the *Results* section[Sec sec1] have shown *MapsTorch*’s robust performance for tasks like first-pass fitting without good initialization and elemental refinement in ROIs across large scale historical datasets.

## 3. Results

### 3.1. Experiment setup

To evaluate the performance of *MapsTorch* for XRF data analysis, we collected approximately 1200 historical XRF datasets from the 2-ID-E beamline at the APS, covering the period from 2012 to 2017. For each dataset, the spectrum data was retrieved along with manually selected target elements and manually tuned parameters in the metadata files written by beamline scientists. This large and diverse benchmark set provides a robust testing bed for assessing fitting quality and robustness under user facility setting for developing automated workflows.

In a typical XRF data analysis workflow, we first fit the integrated spectrum (summed from all individual spectrum at each scanning point/pixel) using a nonlinear optimization algorithm such as *NLopt* to tune the fitting parameters iteratively until a good fit is achieved. Then we fix the fitted parameters and use fast linear optimization algorithms (after fixing all fitting parameters, fitting becomes linear) such as singular value decomposition (SVD) to fit the elemental intensities of all the scanning points/pixels to obtain elemental maps for an initial understanding of the full dataset. Additionally we can identify smaller regions of interest (ROIs) and use an advanced nonlinear optimization algorithm again to get the most accurate elemental intensities in the ROIs for accurate downstream quantification tasks.

Among the three major steps, the second step (*i.e.* linear elemental mapping) can be reliably automated but the first and the last steps (initial spectrum fitting and elemental intensity refinement) are currently manually intensive. We thus focus on these two tasks to evaluate the performance of *MapsTorch* and its potential in enabling automated XRF data analysis workflows. For each task, we compare *MapsTorch* (using *PyTorch*’s default optimizer Adam) with the currently used nonlinear optimization algorithm *NLopt*. The specific *NLopt* implementation is from *XRF-Maps* (Glowacki, 2020 ▸), which is widely used in the APS community.

In the initial parameter optimization task, both the fitting parameters (*e.g.* energy calibration, peak shapes, *etc*.) and the elemental intensities need to be optimized. The information needed in the fitting algorithm are the experimental spectra and the set of target elements. This is the most common task for XRF data analysis, where the analyst has a list of target elements but does not yet have calibrated fitting parameters. Achieving robust and high quality fits in this scenario is critical for enabling automated initial XRF data analysis.

As for the elemental intensity refinement task, the analysts have access to tuned parameters and focus on refining the elemental intensities in ROIs. The fitting parameters may need slight adjustment/tuning to be customized to specific recorded signals in ROIs. This scenario reflects the typical use case of analyzing ROIs with high precision to get most accurate elemental intensities. In practice, this step is often used to confirm trace elements or to accurately quantify low concentration elements in ROIs where standard fast linear fitting algorithms often fail to accurately capture.

All comparisons use a fully automated, no-intervention setup: initial fits start from random parameter and amplitude initializations, ROI refinements start from the integrated spectrum fitted parameters, and no manual adjustments are made between iterations.

### 3.2. Result discussion

**Figure 2:**
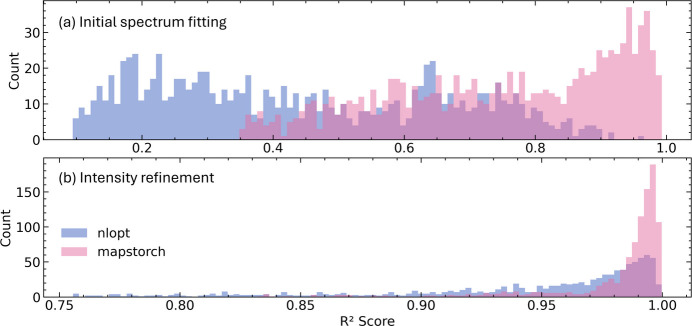
Histogram of *R*^2^ across ∼1200 datasets for two spectrum fitting tasks. Top: initial spectrum fitting task (random parameter initialization) results. Bottom: elemental amplitude refinement task (further fitting with parameters initialized to fitted values) results. In each subplot, *MapsTorch* (in pink color, AD + Adam approach) and *NLopt* (in purple color, finite difference + traditional nonlinear optimizer approach) *R*^2^ histograms are compared.

For a comprehensive view of performance comparison between *MapsTorch* and *NLopt*, we present histograms showing how the *R*^2^ values are distributed across all datasets in Fig. 2 ▸ and report distribution statistics in Table 5 ▸. We note that all comparisons between *MapsTorch* and *NLopt* for the same dataset use the same set of target elements and the same set of fitting parameters. The only differences between *NLopt* and *MapsTorch* fitting runs of the same dataset are the fitting mechanics: AD method and Adam optimizer in *MapsTorch* versus finite difference method and traditional nonlinear optimization algorithm in *NLopt*.

For the initial spectrum fitting task, the histograms in Fig. 2 ▸(*a*) show that the *MapsTorch* fitting quality distribution is concentrated toward higher *R*^2^ values, with a large portion of datasets achieving above 0.8 and many above 0.9. In contrast, *NLopt* exhibits a noticeable cluster of lower scoring fits. This shows the robustness of the *MapsTorch* fitting quality without good initializations, under the scale and diversity of synchrotron datasets. This robust performance of *MapsTorch* can be attributed to its advanced AD-based optimizer, which can effectively navigate the parameter search space. This result is especially relevant to processing new or unfamiliar samples’ data with minimal prior knowledge (*i.e.* only list of target elements). With *MapsTorch*’s performance, it is now possible to obtain relatively good fits without iterative manual tuning, which can enable future automated initial data processing and screening workflows.

**Figure 3:**
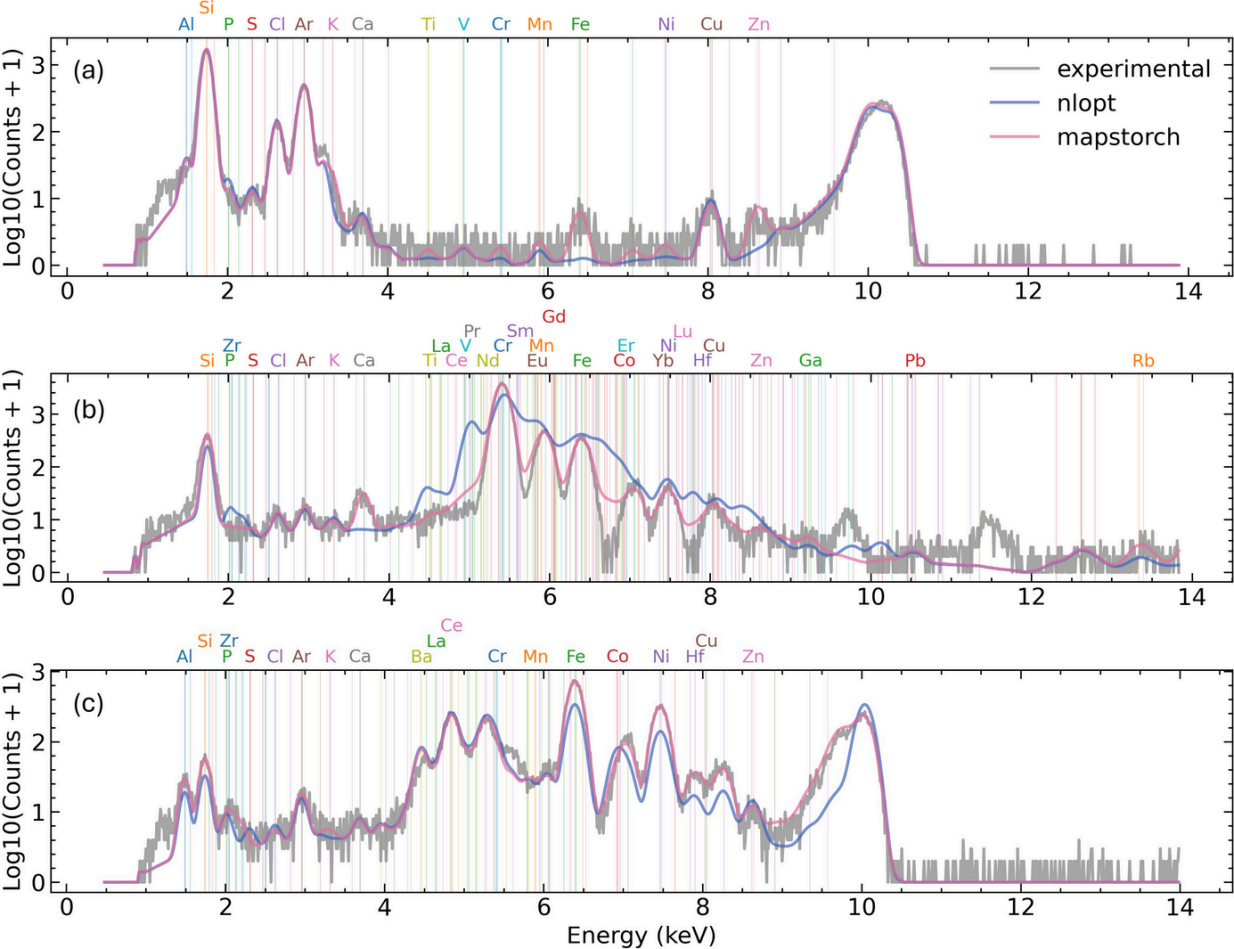
Representative examples from the elemental intensity refinement task shown in Fig. 2 ▸(*b*). In each subplot (*a*)–(*c*), gray shows the experimental spectrum on a log scale, purple and pink curves are the *NLopt* and *MapsTorch* fits, respectively, using the same element list and parameter initialization. Spectra are shown as log_10_(counts+1) versus energy (keV). Colored vertical ticks label the characteristic line energies of the fitted elements. Across all three low intensity spectra, *MapsTorch* can fit low intensity spectra with small peaks better and yield better fits than *NLopt*.

For the elemental intensity refinement task, the fitting parameters are initialized to previously fitted values; the fitting parameters and elemental amplitudes will be further optimized in the fitting algorithm. We observe in Fig. 2 ▸(*b*) that both *MapsTorch* and *NLopt* achieve higher *R*^2^ values overall as expected. Nevertheless, *MapsTorch* remains more sharply peaked near higher *R*^2^ scores, reflecting an advantage in pushing the fitting quality further and capturing subtle spectral features. This is especially relevant for low intensity spectra where accurate amplitude fitting for low intensity peaks can be challenging for traditional fitting methods. To highlight this, we show some *MapsTorch* and *NLopt* fitting results for low intensity spectra with weak peaks in Fig. 3 ▸. We observe that *NLopt*, at its algorithmic convergence, can miss some small peaks, and can result in overall suboptimal fitting quality. In contrast, *MapsTorch* can effectively fit those low intensity spectra with weak peaks.

The performance difference in the elemental intensity refinement task can be explained by the different optimization mechanics. In *NLopt*, traditional nonlinear optimization algorithms can terminate near a plateau once the objective, mostly driven by the large-amplitude portions of the spectrum, ceases to improve beyond a tolerance, thus leaving low count lines underfit. In contrast, Adam can continue to receive informative gradients from small peak regions and perform coordinated small updates, so weak lines’ fits keep improving. We note that if analysts fit those low intensity spectrum examples manually, they should be able to identify missing peaks and re-fit specific regions, but such manual effort cannot be automated and scaled. Now with the robust performance of *MapsTorch* on low intensity spectra, it is possible to develop automated workflows that can avoid such manual efforts.

In summary, across both tasks, *MapsTorch* shows higher quality fits than *NLopt*, which can be attributed to the AD technique coupled with the modern AD-based optimizer being less sensitive to initialization and better at exploiting subtle information from data. Practically, these results imply the possibility to replace the two most labor-intensive bottlenecks in XRF data analysis: the initial spectrum fitting and the elemental intensity refinement. We note that when parameters are already close to optimal (for example, reused from similar samples on the same instrument), *NLopt* typically delivers good fits. Thus in routine operations where expert-tuned parameter files are already available, *NLopt* remains effective. The advantage of *MapsTorch* is its resilience to poor initialization and its ability to keep improving weak lines without manual intervention. The contribution here is to enable automated processing when such retuning would otherwise be required repeatedly. For example, in a future end-to-end automated XRF data analysis workflow, *MapsTorch*’s‘first pass’ fitting can produce reliable initial fits with only a list of target elements input, then, after the linear elemental mapping and ROI identification, the same *MapsTorch* engine can act as a refinement tool to push ROIs’ fit to higher accuracy, improving downstream tasks such as trace elements identification and elemental quantification. Overall the observed robust performance of *MapsTorch* can be translated into benefits in automating XRF data analysis, reducing human efforts and bias, and improving analysis consistency and reproducibility.

### 3.3. Case study: automated first-pass and ROI refinement

To illustrate how *MapsTorch* can help in automating XRF data analysis, we present a case study on an example plant sample dataset acquired at the 2-ID-E beamline at APS. The raster scan comprises ∼500 × 600 spectra, also yielding per-pixel spectrum and integrated spectrum. The target element list was specified by a beamline scientist and remained the same in all runs.

**Figure 4:**
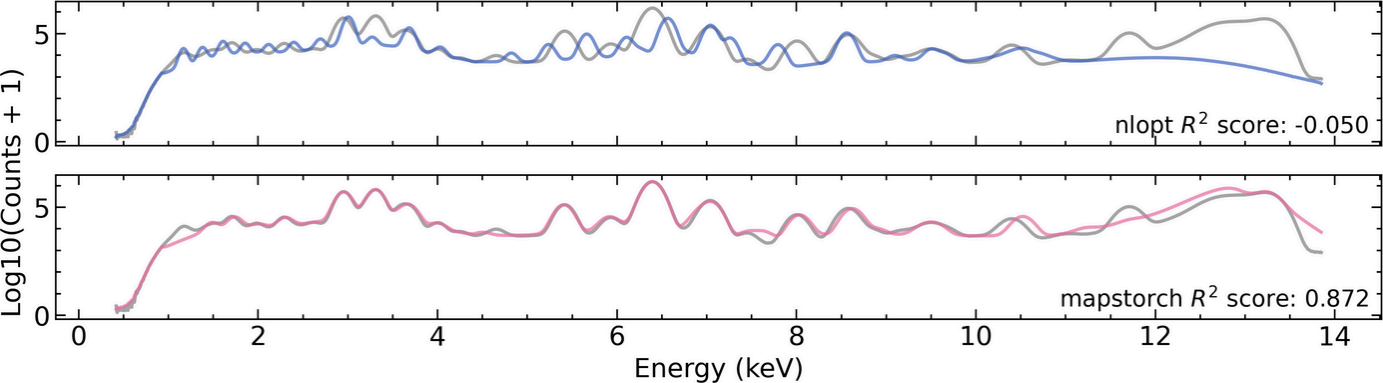
Case study: first-pass fit of the integrated spectrum from a plant sample. Both methods start from random parameter and amplitude initialization (within reasonable ranges) with no manual adjustments and use the same target element list. Top: *NLopt* result (finite-difference gradients with a traditional nonlinear optimizer) stalls far from the data (*R*^2^ = −0.050). Bottom: *MapsTorch* (automatic differentiation with Adam optimizer) reaches a substantially better first-pass fit (*R*^2^ = 0.872). Spectra are shown as log_10_(counts+1) versus energy (keV).

We first compared the first-pass spectrum fitting performance. We initialized all fitting parameters and all elemental amplitudes with random values (sampled within reasonable ranges), then fit the integrated spectrum. As shown in Fig. 4 ▸, *MapsTorch* achieved acceptable first-pass fitting quality (*R*^2^ = 0.872) after 500 Adam optimization steps. On the contrary, *NLopt* only results in *R*^2^ = −0.05 at convergence.

**Figure 5:**
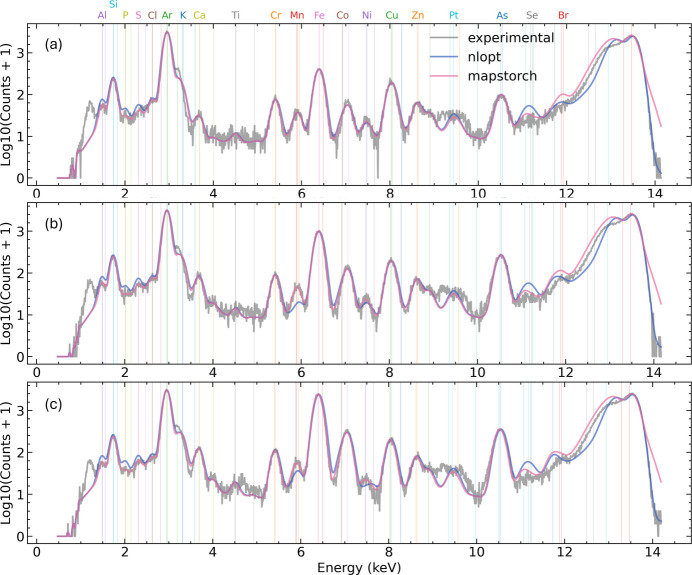
Case study: ROI elemental intensity refinement. Subplots (*a*)–(*c*) show fitting results in three 25 × 25 sized regions of interest. Parameters are initialized to the values obtained from the integrated spectrum fitting and then refined together with elemental amplitudes. Spectra are shown as log_10_(counts+1) versus energy (keV). Gray denotes the experimental spectra, purple and pink are the *NLopt* and *MapsTorch* fits. Vertical colored markers indicate the characteristic lines of the fitted elements. *MapsTorch* consistently reduces residuals at weak lines (*e.g.* Al, S, Mn, Ni), resulting in higher-quality fits.

Next we compared the elemental intensity refinement performance. We initialize all fitting parameters to previously fitted good ones and ran the fitting algorithms again on three different 25 × 25 sized ROIs. As seen from Fig. 5 ▸, *NLopt* cannot accurately fit small peaks (*i.e.* Al, P, S, Mn, Ni) while *MapsTorch* can fit those weak peaks better and push the fitting quality further. The accurate fitting of those weak lines matters for downstream tasks such as trace element identification and quantification. We note that in traditional XRF data analysis it is possible for experts to isolate those weak lines and re-fit manually, but this manual process cannot be scaled to automated pipelines. On the contrary, *MapsTorch*, owing to its better optimization mechanics, can fit those low intensity peaks better, thus can reduce or even avoid manual refinements.

**Figure 6:**
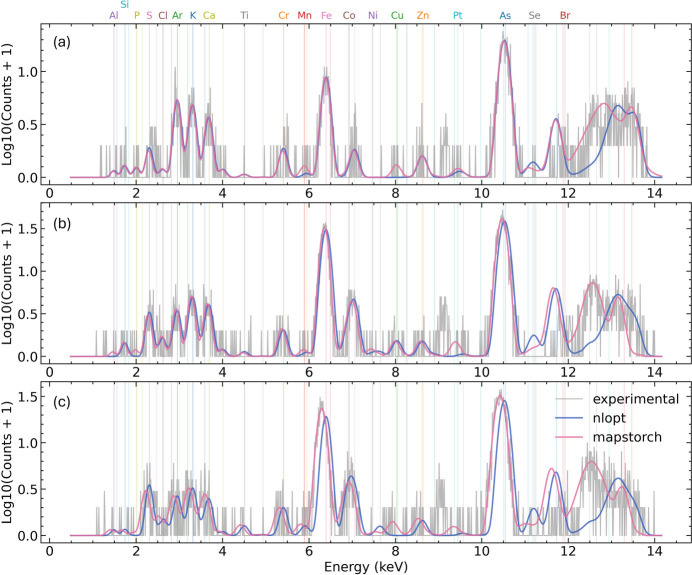
Case study extension: single pixel spectrum refinement. Three representative pixels (*a*)–(*c*) highlight fitting behavior at very low photon count levels. Using the same parameter initialization from the integrated spectrum fitting, *MapsTorch* (pink) remains stable and captures weak peaks (*e.g.* Mn near 6 keV, Cu/Zn near 8 keV and Pt *L* lines around 10 keV) well, whereas *NLopt* (purple) under-fits or results in suboptimal fitting quality. These examples reflect the same no-intervention automated setup described in Section 3.3. Spectra are shown as log_10_(counts+1) versus energy (keV).

**Figure 7:**
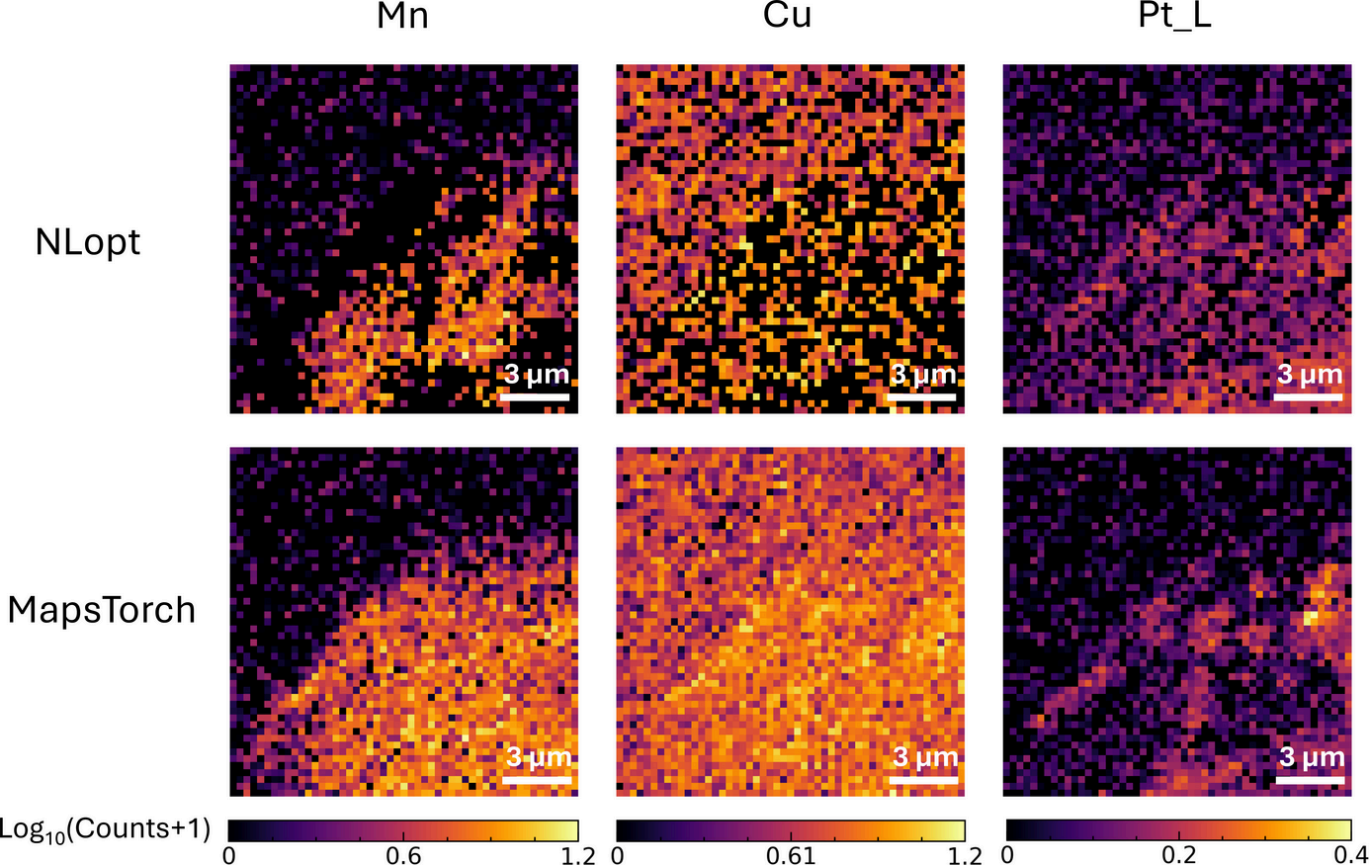
Case study extension: elemental maps derived from per-pixel nonlinear fits. Columns show Mn, Cu and Pt *L* amplitudes, the top row is *NLopt* and the bottom row is *MapsTorch*. *MapsTorch* yields smoother, more coherent, spatial structure without artifacts for Mn and Cu, and reveals features that are muted in *NLopt* maps for Pt *L*. The improved elemental maps are consistent with the better single-pixel fits in Fig. 6 ▸.

Furthermore, we observed that the robust performance of *MapsTorch* can even be pushed to single pixel spectrum fitting. For example, in Fig. 6 ▸ we observe that *MapsTorch* achieved stable fitting performance across single pixels’ spectra with very low intensity. On the contrary, *NLopt* under-fitted some lines (*i.e.* Mn, Cu, Pt) and sometimes was completely off. We further plotted those elements’ elemental maps in Fig. 7 ▸ and observed that, owing to the inaccurate fittings from *NLopt*, the elemental maps may show artifacts (*i.e.* Mn and Cu) or miss spatial features (*i.e.* Pt). In typical XRF data analysis, we usually assume that all pixels share the same set of fitting parameters, so we can transform elemental mapping into a linear problem by fixing all fitting parameters to integrated spectrum fitted values, such that fast linear solvers can be used to generate elemental maps. Trading off the efficiency and accuracy, it is often not necessary to use slower nonlinear optimization to fit each pixel. This per pixel spectrum fitting test and results here are just to demonstrate the robustness of *MapsTorch* and potential new possibilities that can be enabled by *MapsTorch*. We note that all case-study runs follow the same no-intervention automated processing conditions. Besides, results shown in Figs. 6 ▸ and 7 ▸ reflect the extreme case of single pixel spectrum fitting at very low photon counts. Thus the poor performance of *NLopt* here does not invalidate prior expert-tuned *NLopt* fitting results.

All comparisons above use Python scripts without manual intervention. The robust performance of *MapsTorch* means that it is possible to create a single script to complete: (1) a first-pass fit of the integrated spectrum using *MapsTorch*, (2) quick per-pixel amplitude fitting with linear solvers, (3) ROIs identification, (4) ROI elemental intensity refinement using *MapsTorch*. The equivalent data processing pipeline using *NLopt* would require good initialization of fitting parameters, iterative manual adjustment of parameters, low intensity peak isolation and re-fitting, *etc*., which is time-consuming and prone to human bias.

## 4. Conclusion and future directions

We developed the *MapsTorch* package that implements classic *MAPS* analytical models in *PyTorch* for XRF spectrum analysis. This differentiable implementation enables the efficient gradient calculation method AD and unlocks advanced optimizers such as Adam for spectrum fitting. Our computational experiments on large numbers of diverse historical synchrotron datasets show the robust performance of *MapsTorch* in tasks such as initial spectrum fitting and elemental intensity refinement, which are traditionally manually intensive. Consequently, *MapsTorch* has great potential in reducing human effort and enabling fully autonomous XRF data analysis workflows. We note that *MapsTorch* is intended to complement, not replace, expert-driven *NLopt* workflows. When well tuned parameter files exist, traditional pipelines remain effective. The contribution here is to make automated processing reliable when repeated manual retuning would otherwise be necessary.

There are several interesting avenues for further development of *MapsTorch* and the AD approach for XRF analysis. One area is the development of more sophisticated physical models for the XRF spectrum. While the current implementation in *MapsTorch* covers many important aspects of XRF spectroscopy, there is room for improvement in areas such as accounting for self-absorption effects and handling overlapping peaks from different elements. Furthermore, it is possible to integrate other spectroscopic or imaging techniques in a multi-modal data analysis framework. The flexible AD approach makes it straightforward to implement and test new models or constraints without explicitly calculating gradients and modifying the optimization loop.

Integration with machine learning models is another promising area for future development. The differentiable nature of *MapsTorch* makes it well suited for incorporation into larger neural network architectures. This could enable the development of hybrid models that combine physics-based XRF spectrum modeling with data-driven approaches, potentially leading to more robust and generalizable analysis methods. For example, one could envision a neural network that learns to predict material properties based on raw spectral data, using the *MapsTorch* models as a differentiable layer in the network.

Another important direction is uncertainty quantification and Bayesian inference. By leveraging recent advances in AD-based probabilistic programming frameworks, *MapsTorch* could be extended to provide not just point estimates of fitting parameters and elemental intensities but also full posterior distributions. This would allow researchers to better understand the uncertainty in their results and make more informed decisions about experimental design and data interpretation.

It is important to acknowledge the limitations of the AD approach. The reliance on gradient-based optimization means that *MapsTorch*’s performance is still upper bounded by the objective function. Besides, the accuracy of the AD approach is fundamentally limited by the accuracy of the underlying physical models used to describe the XRF process. Inaccuracies or simplifications in these models can propagate through the analysis. While there are challenges and limitations to be addressed, the future directions outlined here suggest that the AD approach has the potential to elevate not only XRF analysis but also a wide range of spectrum-based analytical techniques.

## Figures and Tables

**Table 1:** Pseudo-code for 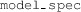 function

**Function:** 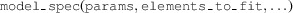
1: Initialize model spectrum
2: **For** each element in elements_to_fit:
3: Calculate element spectrum using 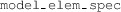
4: Add element spectrum to model spectrum
5: Calculate elastic spectrum using 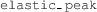
6: Calculate Compton spectrum using 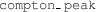
7: Add elastic and Compton spectra to model spectrum
8: **If** escape peaks are enabled:
9: Calculate escape peaks using 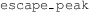 and add to model spectrum
10: **Return** model spectrum

**Table 2:** Pseudo-code for 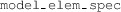 subroutine

**Function:** 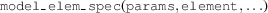
1: Initialize element spectrum
2: Get energies, branching ratios, 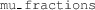 *etc*. for input element
3: Calculate binding energies for each line
4: Determine valid lines based on binding energies and incident energy
5: Calculate sigma (peak width) for each line
6: Calculate 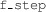 and 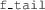 factors
7: Calculate 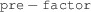 and 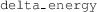 for each line
8: **For** each line:
9: **If** line is valid:
10: Calculate peak intensity using Gaussian function
11: Add step contribution to peak
12: Add tail contribution to peak
13: Add modified peak to element spectrum
14: Apply corrections to element spectrum
15: **Return** spectrum

**Table 3:** Pseudo-code for 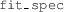 function

**Function:** 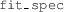 ( 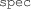 , 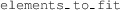 , 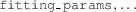 )
1: Create tensors for parameters and data using 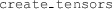 function
2: **For** iteration in 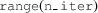 :
3: Zero out gradients
4: Calculate background using 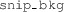 function
5: Calculate model spectrum using 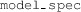 function
6: Calculate fitting quality using loss function
7: Backpropagate loss value to calculate gradients
8: Update parameters and/or amplitudes using gradient-based optimizer
9: **Return** 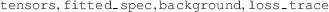

**Table 4:** Pseudo-code for 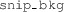 function

**Function:** 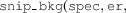 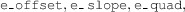 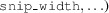
1: Extract bounds from er and map channels to energy using calibration parameters
2: Estimate per-channel width from the energy resolution model
3: Initialize background by smoothing the spectrum with a boxcar filter
4: Stabilize background values using a logarithmic transform
5: Determine valid index bounds and initialize clipping width
6: Apply inlined SNIP clipping a few times
7: **While** the clipping width remains large: repeat SNIP clipping and gradually reduce the width
8: Restore original scale by inverting the stabilization transform
9: Clean numerical artifacts in the background
10: **Return** background

**Table 5:** Summary statistics of *R*^2^ across ∼1200 datasets

Task	Method	Mean ± s.t.d.	Median	% *R*^2^ > 0.7	% *R*^2^ > 0.8	% *R*^2^ > 0.9
Initial spectrum fitting	*MapsTorch*	0.66 ± 0.27	0.72	52.32	38.81	24.10
Initial spectrum fitting	*NLopt*	0.39 ± 0.26	0.35	14.46	4.22	0.34
Intensity refinement	*MapsTorch*	0.93 ± 0.19	0.99	87.01	85.89	82.36
Intensity refinement	*NLopt*	0.88 ± 0.20	0.96	88.12	81.24	69.28

## Data Availability

*MapsTorch* is open-source software and can be accessed at https://github.com/AdvancedPhotonSource/MapsTorch. Users can test the methodology and software using a demo dataset at https://huggingface.co/spaces/shawnyin/*MapsTorch*.

## Footnotes

1 https://www.silx.org/doc/PyMca/dev/training/quantification/index.html, https://nsls-ii.github.io/PyXRF/summed_spectrum_fit.html, https://wiki.classe.cornell.edu/CHESS/Maia/IntroGeoPIXEWorkflowOverview.
